# Factors associated with health-related quality of life in Koreans aged over 50 Years: the fourth and fifth Korea National Health and Nutrition Examination Survey

**DOI:** 10.1186/s12955-017-0816-4

**Published:** 2017-12-16

**Authors:** Kyoung Min Kwon, Jung Soo Lee, Na Eun Jeon, Yeo Hyung Kim

**Affiliations:** Department of Rehabilitation Medicine, College of Medicine, The Catholic University of Korea, Uijeongbu St. Mary’s Hospital, Geumo-dong, Gyeonggi-do, 480-717 Uijeongbu-si Republic of Korea

**Keywords:** Quality of life, Health, Aged

## Abstract

**Background:**

To evaluate and analyse the factors associated with health-related quality of life (HR-QoL) in the Korean population aged 50 years and older.

**Methods:**

We used data obtained from the fourth and fifth Korea National Health and Nutrition Examination Survey (KNHANES IV-V) 2007–2012, a national, cross-sectional health examination and survey, for which representative data on the health, nutritional status, and physical activities of the Korean general population are collected by the Korea Centres for Disease Control and Prevention (KCDC). The sampling protocol for the KNHANES was designed to involve a complex, stratified, multistage probability cluster survey of a representative sample of the noninstitutionalized civilian population in South Korea using a cross-sectional design. The association between HR-QoL and socio-economic factors and medical comorbidities in adults aged 50 years and older was investigated using data from the KNHANES IV-V from 2007 to 2012 (n = 17,937). The EuroQol 5-Dimension Questionnaire (EQ-5D) was used to evaluate HR-QoL, and the factors associated with HR-QoL were analysed after adjusting for socio-economic and demographic factors, anthropometric measurements and clinical comorbidities.

**Results:**

Health status declined with ageing, and low socio-economic status had negative associations with health status. Gender had no association with health status. Among chronic medical conditions, arthritis and depression had significant associations with health status in older people when stratified by age and gender (*P* < 0.05).

**Conclusions:**

These findings suggest that older people may value the ability to perform daily activities, which may mean that it is necessary to pay more attention to the factors associated with musculoskeletal pain and emotional distress, as well as socio-economic status or chronic diseases.

**Trial registration:**

The Korea National Health and Nutrition Examination Survey (KNHNES) is not a clinical trial registry, but the national health survey conducted by the Government of the Republic of Korea, to gather information on health and nutritional status of Korean population, to plan public health services and health education programs, and to do statistical studies of the efficiency of public health services required for setting up welfare and healthcare policies.

## Background

The individual perception of health status is representative of health-related quality of life (HR-QoL). HR-QoL is indicative of an individual’s subjective evaluation of his own well-being and is a useful tool for predicting the mortality of a population group and serves as an outcome measure for the economic evaluation of healthcare services [1, 2]. As the population ages, the prevalence of chronic diseases increases, but in the past several decades, the developments of clinical interventions and prevention measures have reduced the age-adjusted mortality rate and increased the life expectancy of older adults. The quality of life of older people has been emphasized.

Quality of life (QoL) has been defined by the World Health Organization (WHO) as ‘individuals’ perception of their position in life according to their purposes, expectations, standards and worries within the context of the culture and value system in which they live’. In a connected concept, HR-QoL is a construct that focuses on individuals’ subjective perception of health status and consists of physical, mental, and social dimensions [3]. Due to the increasing importance of HR-QoL, several utility measures, such as the EuroQol 5-Dimension Questionnaire (EQ-5D) and the SF-6D (Short Form-6 dimension), have been introduced to assess patient-reported health status. The EQ-5D is a standardized, simple, and generic measure of health perceptions consisting of a descriptive system with five dimensions and an EQ-VAS. The evaluation of the EQ-5D, a useful tool for HR-QoL, was demonstrated [4, 5], and an analytic study that compared the validity of the EQ-5D and the SF-6D in patients with chronic pain suggested that the EQ-5D has higher construct validity and responsiveness than the SF-6D [6].

Previous studies have shown that several factors, including age, sex, functional status, socio-economic status, psychomotor impairment, and medical comorbidities, influence HR-QoL in particular disease groups [7–9]. A study on coronary heart disease demonstrated that the predictors for low self-rated health status were low income and non-cardiovascular comorbidities [10]. A multicentre prospective cohort study using interviews from individuals aged 75 years and over in Germany showed that social engagement had a positive impact on HR-QoL and decreased depressive symptoms in the total sample and in women [11]. A population-based study of adults (18 to 65 years) living in Brazil showed that depression had the greatest impact on HR-QoL, and social class, employment status, and place of residence also affected HR-QoL [12].

However, older people suffer from not one but many different underlying medical conditions. HR-QoL may be influenced by a complex interplay among various factors, such as socio-economic status, anthropometric characteristics or medical comorbidities, rather than by one particular factor, which is supported by the findings of an Irish cohort study [13]. This study demonstrated that QoL is determined by a number of life domains, rather than one sole dominating domain. Even though physical health becomes poor, QoL can often remain high if individuals maintain good status in other domains of life. Unfortunately, there are far fewer studies that have examined the multiple factors affecting HR-QoL, using data representing the prevalence of chronic diseases in the general elderly population.

The aim of this study is to investigate the factors associated with QoL using data from the population aged 50 and older and to identify whether there are other factors that differ from those of previous studies.

## Methods

### Study design and participants

This study was based on data obtained from the fourth and fifth Korean National Health and Nutrition Examination Survey (KNHANES IV-V), 2007–2012 in the Republic of Korea (hereafter ‘Korea’). The KNHANES is a national, cross-sectional health examination and adopts a rolling sampling survey to represent the probability sampling of health, nutritional status, and physical activities in the Korean general population. The sampling protocol for the KNHANES was designed to involve a complex, stratified, multistage probability cluster survey of a representative sample of the noninstitutionalized civilian population in Korea. Each survey year includes a new, different sample of approximately 10,000 individuals aged 1 year and over by systematic sampling. This study included 17,937 participants aged over 50 years from 2007 to 2012, because the prevalence of chronic disease is rising for individuals over 50 years of age. The overall response rate of participants was 78.4% in KNHANES IV and 80% in KNHANES V. Informed consents to participate in the study were obtained from all participants before they participated in the KNHANES.

### Dependent variable

HR-QoL was measured using the EQ-5D. In the KNHANES, the participants were asked to choose one of the following three responses for each of the five given dimensions that best explained their current health status: 1 = “no problem,” 2 = “some problems”, and 3 = “severe problems”. The five questions concerning health status expressed health status between 1, which represents perfect health status, and −1, which represents a health status that is no better than death. In this study, the EQ-5D index, which Nam et al. calculated using their estimated weighted quality value for Koreans, was used [14]. The formula for the EQ-5D index is as follows: EQ-5D index = 1 - (0.05 + 0.096 × M2 + 0.418 × M3 + 0.046 × SC2 + 0.136 × SC3 + 0.051 × UA2 + 0.208 × UA3 + 0.037 × PD2 + 0.151 × PD3 + 0.043 × AD2 + 0.158 × AD3 + 0.05 × N3), where M2 - mobility “level 2” = 1; otherwise, 0; M3 - mobility “level 3” = 1; otherwise, 0; SC2 - self-care “level 2” = 1; otherwise, 0; SC3 - self-care “level 3” = 1; otherwise, 0; UA2 - usual activities “level 2” = 1; otherwise, 0; UA3 - usual activities “level 3” = 1; otherwise, 0; PD2 - pain/discomfort “level 2” = 1; otherwise, 0; PD3 - pain/ discomfort “level 3” = 1; otherwise, 0; AD2 - anxiety/depression “level 2” = 1; otherwise, 0; AD3 - anxiety/depression “level 3” = 1; otherwise, 0; N3 - only one “level 3” = 1, and the rest = 0.

### Independent variables

Gender was categorized into male and female, and age was divided into three groups: age 50–59, age 60–69, and over age 70. Body mass index (BMI, kg/m^2^) was categorized into low weight (< 18.5 kg/m^2^), normal weight (18.5 ≤ BMI < 25 kg/m^2^), and overweight (25 kg/m^2^ ≤ BMI).

Residential area, marital status, and socio-economic status variables were collected by interviewing participants, which was performed by trained staff using a standardized form.

The location of residence and marital status were each categorized into two groups: rural vs. urban area (classified by administrative district) and married vs. unmarried (including divorced, widow or widower)*.* We focused on three dimensions of socio-economic status (i.e., educational level, household net income, employed status). Educational level was categorized into four groups: education for 6 years or less as elementary school; 7–9 years as middle school; 10–12 years as high school; and 13 years or over as college or university. Total household net income was categorized into quartiles from Q1 (low) to Q4 (high), based on average household monthly net income. Smoking status was also included, which was categorized into current smoker vs. ex-smoker or non-smoker.

Underlying medical conditions included diabetes mellitus (DM), hypertension (HBP), arthritis (including osteoarthritis or/and rheumatoid arthritis with pain symptoms), coronary artery disease (CAD), chronic renal disease (CRD), cancer, dyslipidaemia, stroke and depression, because these medical comorbidities are most commonly encountered in clinics. Arthritis, CAD (including myocardial infarction or angina pectoris), CRD, dyslipidaemia, stroke and depression were defined as when participants suffered from the disease under a physician’s diagnosis or/and took medications at the time of self-reporting. Cancer was defined as when participants suffered from one of the following cancers: gastric, colon, breast, lung, cervix or uterus, thyroid, and liver, and the cancers were under treatment or in complete remission at the time of self-reporting. They all were classified into two groups according to a “yes or no” questionnaire.

DM was defined as a serum fasting glucose level ≥ 126 mg/dl, use of anti-diabetic medications, or a physician’s diagnosis of DM, and impaired fasting glucose was defined as a fasting plasma glucose level of 100–125 mg/dl, which were dichotomized as having DM. Fasting blood glucose level was measured by an enzymatic method. HBP was defined as systolic blood pressure ≥ 140 mmHg, diastolic blood pressure ≥ 90 mmHg, or use of anti-hypertensive medications, which were also dichotomized as having HBP. The blood pressure was measured by trained medical staff following a standardized procedure during the health examination survey.

### Statistical analysis

The collected raw data of the KNHANES are not the complete enumeration survey but rather the sample survey with complex sampling design, which means that sample data do not have equal probability of being selected. It is recommended that sampling weights are used to analyse the data and estimate results for the target population. The sampling weight was calculated from sampling weights, non-response adjusted weights using estimated response probability, and calibration weights, taking into account the clustering and stratification of the sample survey data.

We adopted complex-samples descriptive methods, complex-samples general linear model (CSGLM) or complex-samples logistic regression model (CSLRM) to analyse collected raw data, as recommended in the user guidelines of the KNHANES. Multicollinearity among the investigated variables was assessed, and final multiple linear and logistic regression model with the selected covariates was computed.

*P* values <0.05 were considered to be statistically significant. Data are presented as estimated mean percentages ± standard error (with 95% confidence intervals [CI]) for categorical variables and estimated mean ± standard error (with 95% CI) for continuous variables. All statistics were calculated using Statistical Package for the Social Sciences version 22 (IBM/SPSS Inc., Armonk, NY, USA).

## Results

### General characteristics of the participants

A total of 17,937 participants aged over 50 years were included, consisting of 7627 males and 10,310 females. The mean score of the EQ-5D index in the 17,937 participants was estimated as 0.90 ± 0.00 (95% CI: 0.89–0.90). The mean score of the EQ-5D index in male group (*n* = 7627) was 0.93 ± 0.00, which was higher than in the female group (*n* = 10,310, 0.87 ± 0.00), without adjusting for covariates.

Both the male and female group had a higher proportion of participants in the 50–59 age group and with normal BMI, elementary educational level and low or low to mid quartile household net income levels. The proportion of current marital status, employed status, and smokers was significantly higher in the male group (*P* < 0.05). The prevalence of DM, HBP, CAD and stroke was higher in male group, but that of dyslipidaemia, arthritis, cancer and depression was higher in the female group (*P* < 0.05). However, the prevalence of CRD and the residential areas was not different between the two genders. The general characteristics of the 17,937 participants were summarized in Table 1.

**Table 1:** General characteristics of the 17,937 participants aged over 50 years

	Total (*n* = 17,937)	Male (*n* = 7627)	Female (*n* = 10,310)	*P*-values
EQ-5D index	0.90 ± 0.00	0.93 ± 0.00	0.87 ± 0.00	0.00^†^*
Age (n (%))				
50–59 years	6441 (46.8%)	2733 (50.6%)	3708 (43.5%)	0.00^‡^*
60–69 years	5936 (28.2%)	2619 (28.9%)	3317 (27.7%)	
70+ years	5560 (25%)	2275 (20.6%)	3285 (28.8%)	
BMI (n (%))				
Low	563 (2.8%)	296 (3.3%)	267 (2.4%)	0.00^‡^*
Normal	10,986 (61.3%)	4904 (63.4%)	6082 (59.5%)	
Obesity	6291 (35.9%)	2381 (33.4%)	3910 (38.1%)	
Educational level (n (%))				
Elementary school	9165 (48.5%)	2682 (33.0%)	6483 (61.9%)	0.00^‡^*
Middle school	2895 (18.2%)	1453 (20.8%)	1442 (15.9%)	
High school	3548 (22.4%)	1979 (28.6%)	1569 (17.0%)	
College	1802 (10.9%)	1278 (17.5%)	524 (5.2%)	
Household net income (n (%))				
Low	5912 (29.6%)	2212 (25%)	3700 (33.7%)	0.00^‡^*
Low to mid	4579 (26.3%)	1988 (26.3%)	2591 (26.4%)	
Mid to high	3437 (21.1%)	1560 (22.6%)	1877 (19.9%)	
High	3552 (22.9%)	1683 (26.2%)	1869 (20.1%)	
Residential area, City (n (%))	7435 (44.6%)	3154 (44.7%)	4281 (44.6%)	0.94^‡^
Current marital status, married (n (%))	13,495 (77.3%)	6918 (91.9%)	6577 (64.9%)	0.00^‡^*
Employed (n (%))	8700 (53.7%)	4679 (68.1%)	4021 (41.3%)	0.00^‡^*
Smoking (n (%))	2822 (19.2%)	2392 (35.8%)	430 (4.9%)	0.00^‡^*
DM (n (%))	6631 (41.7%)	3246 (46.9%)	3385 (37.2%)	0.00^‡^*
HBP (n (%))	13,073 (74.2%)	5683 (76.2%)	7390 (72.5%)	0.00^‡^*
CAD (n (%))	885 (4.7%)	431 (5.1%)	454 (4.3%)	0.04^‡^*
Dyslipidaemia (n (%))	2733 (15.5%)	939 (13%)	1794 (17.7%)	0.00^‡^*
Stroke (n (%))	726 (3.7%)	368 (4.1%)	358 (3.3%)	0.02^‡^*
CRD (n (%))	126 (0.7%)	47 (0.6%)	79 (0.8%)	0.24^‡^
Arthritis (n (%))	5384 (27.9%)	1113 (13.6%)	4271 (40.2%)	0.00^‡^*
Cancer (n (%))	939 (21.9%)	378 (19.4%)	561 (24%)	0.00^‡^*
Depression (n (%))	3036 (16.6%)	689 (9.3%)	2347 (22.8%)	0.00^‡^*

Values (n (%)) are the number and the percentage of participants

^†^T-test/^‡^Chi-square test and *P*-value (*P < 0.05) for group differences between the male and female group

DM: diabetes mellitus; HBP: hypertension; CAD: coronary artery disease (including myocardial infarction or angina pectoris); CRD: chronic renal disease

### Multivariate-adjusted score of the EuroQol 5-dimension (EQ-5D) index according to ageing

In the adults aged over 50 years, the covariates associated with factors affecting the score of the EQ-5D index were age, BMI, educational level, household net income, residential area, current marital status, employed status, stroke, arthritis, and depression (*P* < 0.05). However, the EQ-5D index was not different between genders, when stratified by age.

To examine the association between modifiable factors and the score on the EQ-5D index, scores were stratified by gender and age. In the male group, age over 70 years, lower educational level (elementary < middle and high < college), lower household net income (low < low to mid and high < mid to high quartiles), CRD, arthritis, cancer, and depression were the factors associated with lowering the score of the EQ-5D index, but BMI, residential area, current marital status, employed status, smoking, DM, HBP, CAD, dyslipidaemia, and stoke were not. In the female group, age over 70 years, low or high BMI, lower educational level (elementary < middle < high and college), lower household net income (low < low to mid < high < mid to high quartiles), unmarried status, unemployment, arthritis, and depression were the factors associated with lowering the score of the EQ-5D index. For both genders, the EQ-5D index tended to significantly decrease according to ageing, lower educational level (elementary < middle and high < college), lower household income (low < low to mid and high < mid to high quartiles), arthritis, and depression (see details in Table 2). Educational level, arthritis and depression were associated with lowering the score of the EQ-5D index in all age groups, when stratified by age. In the 50–59 years age group, household net income and dyslipidaemia were associated with lowering the score of the EQ-5D index. In the 60–69 years age group, BMI, household net income and employed status were associated with lowering the score of the EQ-5D index. In the over 70 years age group, residential area, marital status, employed status, cancer and stroke were associated with lowering the score of the EQ-5D index. With ageing, there were different characteristics of factors associated with lowering the EQ-5D (see details in Table 3).

**Table 2:** Multivariate-adjusted score of EQ-5D index in adults aged over 50 years

	Total		Male		Female	
	Mean ± SE	*P*-values	Mean ± SE	*P*-values	Mean ± SE	*P*-values
Gender						
Male	0.81 ± 0.03	0.54				
Female	0.81 ± 0.03					
Age (years)						
50–59	0.83 ± 0.03	0.00*	0.78 ± 0.04	0.00*	0.84 ± 0.03	0.00*
60–69	0.83 ± 0.03		0.78 ± 0.04		0.84 ± 0.03	
70+	0.77 ± 0.03		0.74 ± 0.04		0.77 ± 0.03	
BMI						
Normal	0.83 ± 0.03	0.00*	0.77 ± 0.04	0.62	0.85 ± 0.03	0.00*
Low	0.79 ± 0.03		0.74 ± 0.05		0.80 ± 0.04	
Obesity	0.81 ± 0.02		0.77 ± 0.04		0.81 ± 0.03	
Educational level						
Elementary	0.78 ± 0.03	0.00*	0.73 ± 0.04	0.00*	0.80 ± 0.03	0.00*
Middle	0.81 ± 0.03		0.77 ± 0.04		0.82 ± 0.03	
High	0.81 ± 0.03		0.77 ± 0.04		0.83 ± 0.03	
College	0.83 ± 0.03		0.79 ± 0.04		0.83 ± 0.03	
Household net income						
Low	0.79 ± 0.03	0.00*	0.75 ± 0.04	0.01*	0.79 ± 0.03	0.00*
Low to mid	0.81 ± 0.03		0.76 ± 0.04		0.82 ± 0.03	
Mid to high	0.82 ± 0.03		0.78 ± 0.04		0.83 ± 0.03	
High	0.82 ± 0.03		0.76 ± 0.04		0.84 ± 0.03	
Residential area						
City	0.81 ± 0.03	0.048*	0.77 ± 0.04	0.11	0.82 ± 0.03	0.25
Rural	0.80 ± 0.03		0.76 ± 0.04		0.82 ± 0.03	
Current marital status						
Married	0.82 ± 0.03	0.01*	0.77 ± 0.04	0.44	0.83 ± 0.03	0.01*
Unmarried, divorced, widow or widower	0.80 ± 0.03		0.76 ± 0.04		0.81 ± 0.03	
Employed						
Yes	0.82 ± 0.03	0.00*	0.77 ± 0.04	0.22	0.83 ± 0.03	0.00*
No	0.8 ± 0.03		0.76 ± 0.04		0.8 ± 0.03	
Smoking						
No	0.81 ± 0.03	0.57	0.76 ± 0.04	0.79	0.83 ± 0.03	0.37
Yes	0.81 ± 0.03		0.76 ± 0.04		0.8 ± 0.04	
DM						
No	0.81 ± 0.03	0.76	0.77 ± 0.04	0.32	0.82 ± 0.03	0.70
Yes	0.81 ± 0.03		0.76 ± 0.04		0.82 ± 0.03	
HBP						
No	0.81 ± 0.03	0.75	0.76 ± 0.04	0.37	0.82 ± 0.03	0.85
Yes	0.81 ± 0.03		0.77 ± 0.04		0.82 ± 0.03	
CAD						
No	0.81 ± 0.03	0.79	0.77 ± 0.04	0.79	0.82 ± 0.03	0.71
Yes	0.81 ± 0.03		0.76 ± 0.04		0.82 ± 0.03	
Dyslipidaemia						
No	0.81 ± 0.03	0.51	0.76 ± 0.04	0.90	0.82 ± 0.03	0.60
Yes	0.81 ± 0.03		0.76 ± 0.04		0.82 ± 0.03	
Stroke						
No	0.83 ± 0.02	0.02*	0.79 ± 0.04	0.05	0.83 ± 0.03	0.15
Yes	0.79 ± 0.03		0.73 ± 0.05		0.8 ± 0.04	
CRD						
No	0.83 ± 0.01	0.30	0.83 ± 0.02	0.04*	0.82 ± 0.02	0.91
Yes	0.79 ± 0.05		0.69 ± 0.07		0.82 ± 0.05	
Arthritis						
No	0.84 ± 0.03	0.00*	0.79 ± 0.04	0.00*	0.86 ± 0.03	0.00*
Yes	0.77 ± 0.03		0.74 ± 0.04		0.78 ± 0.03	
Cancer						
No	0.81 ± 0.03	0.13	0.77 ± 0.04	0.03*	0.82 ± 0.03	0.67
Yes	0.8 ± 0.03		0.75 ± 0.04		0.82 ± 0.03	
Depression						
No	0.83 ± 0.03	0.00*	0.8 ± 0.04	0.00*	0.84 ± 0.03	0.00*
Yes	0.78 ± 0.03		0.73 ± 0.04		0.8 ± 0.03	
R^2^	0.27		0.216		0.275	

*P < 0.05

DM: diabetes mellitus; HBP: hypertension; CAD: coronary artery disease (including myocardial infarction or angina pectoris); CRD: chronic renal disease

**Table 3:** Multivariate-adjusted score of EQ-5D index in adults aged over 50 years according to ageing

	50–59 years		60–69 years		Over 70 years	
	Mean ± SE	*P*-values	Mean ± SE	*P*-values	Mean ± SE	*P*-values
Gender						
Male	0.87 ± 0.04	0.927	0.85 ± 0.02	0.516	0.75 ± 0.05	0.758
Female	0.87 ± 0.04		0.84 ± 0.02		0.75 ± 0.05	
BMI						
Normal	0.89 ± 0.04	0.371	0.87 ± 0.02	0.000*	0.77 ± 0.05	0.223
Low	0.84 ± 0.05		0.85 ± 0.04		0.73 ± 0.06	
Obesity	0.88 ± 0.04		0.82 ± 0.02		0.74 ± 0.05	
Educational level						
Elementary	0.84 ± 0.04	0.000*	0.82 ± 0.02	0.002*	0.70 ± 0.05	0.001*
Middle school	0.87 ± 0.04		0.84 ± 0.02		0.76 ± 0.05	
High school	0.87 ± 0.04		0.85 ± 0.02		0.75 ± 0.05	
College	0.89 ± 0.04		0.86 ± 0.03		0.79 ± 0.05	
Household net income						
Low	0.84 ± 0.04	0.003*	0.82 ± 0.02	0.000*	0.73 ± 0.05	0.063
Low to mid	0.87 ± 0.04		0.84 ± 0.02		0.74 ± 0.05	
Mid to high	0.88 ± 0.04		0.86 ± 0.02		0.74 ± 0.05	
High	0.88 ± 0.04		0.86 ± 0.02		0.78 ± 0.05	
Residential area						
City	0.87 ± 0.04	0.368	0.85 ± 0.02	0.568	0.76 ± 0.05	0.030*
Rural	0.87 ± 0.04		0.84 ± 0.02		0.73 ± 0.05	
Current marital status						
Married	0.87 ± 0.04	0.931	0.86 ± 0.02	0.051	0.77 ± 0.05	0.031*
Unmarried, Divorced, widow or widower	0.87 ± 0.04		0.83 ± 0.03		0.73 ± 0.05	
Employed						
Yes	0.87 ± 0.04	0.636	0.86 ± 0.02	0.002*	0.77 ± 0.05	0.000*
No	0.87 ± 0.04		0.83 ± 0.02		0.72 ± 0.05	
Smoking						
No	0.87 ± 0.04	0.811	0.85 ± 0.02	0.793	0.75 ± 0.05	0.904
Yes	0.87 ± 0.04		0.84 ± 0.03		0.75 ± 0.05	
DM						
No	0.87 ± 0.04	0.589	0.85 ± 0.02	0.649	0.75 ± 0.05	0.612
Yes	0.87 ± 0.04		0.84 ± 0.02		0.74 ± 0.05	
HBP						
No	0.87 ± 0.04	0.800	0.84 ± 0.03	0.406	0.75 ± 0.05	0.933
Yes	0.87 ± 0.04		0.85 ± 0.02		0.75 ± 0.05	
CAD						
No	0.87 ± 0.04	0.89	0.85 ± 0.02	0.512	0.74 ± 0.05	0.421
Yes	0.87 ± 0.04		0.84 ± 0.03		0.76 ± 0.05	
Dyslipidaemia						
No	0.88 ± 0.04	0.023*	0.84 ± 0.02	0.096	0.75 ± 0.05	0.531
Yes	0.86 ± 0.04		0.85 ± 0.02		0.74 ± 0.05	
Stroke						
No	0.87 ± 0.04	0.984	0.86 ± 0.02	0.076	0.79 ± 0.05	0.024*
Yes	0.87 ± 0.04		0.83 ± 0.03		0.70 ± 0.06	
CRD						
No	0.89 ± 0.02	0.627	0.87 ± 0.02	0.264	0.80 ± 0.03	0.094
Yes	0.85 ± 0.07		0.83 ± 0.04		0.70 ± 0.08	
Arthritis						
No	0.90 ± 0.04	0.000*	0.88 ± 0.02	0.000*	0.79 ± 0.05	0.000*
Yes	0.84 ± 0.04		0.81 ± 0.02		0.71 ± 0.05	
Cancer						
No	0.87 ± 0.04	0.752	0.85 ± 0.02	0.259	0.76 ± 0.05	0.013^*^
Yes	0.87 ± 0.04		0.84 ± 0.02		0.73 ± 0.05	
Depression						
No	0.89 ± 0.04	0.000*	0.87 ± 0.02	0.000*	0.78 ± 0.05	0.008*
Yes	0.85 ± 0.04		0.82 ± 0.02		0.72 ± 0.05	
R^2^	0.228		0.232		0.197	

*P < 0.05

DM: diabetes mellitus; HBP: hypertension; CAD: coronary artery disease (including myocardial infarction or angina pectoris); CRD: chronic renal disease

### Comparison of HR-QoL in the lowest quintile and the highest quintile of the EQ-5D

Multivariate-adjusted odds ratios of the lowest quintile group for the highest quintile group of the EQ-5D index in the adults over 50 years of age are shown in Table 4.

**Table 4:** Multivariate-adjusted odds ratios of the lowest quintile of EQ-5D index for the highest quintile in adults aged over 50 years

	Total		Male		Female	
	OR (95% CI)	*P*-value	OR (95% CI)	*P*-value	OR (95% CI)	*P*-value
Gender						
Male (reference)	1	0.05				
Female	1.36 (1–1.85)					
Age(years)						
50–59 (reference)	1	0.00*	1	0.01*	1	0.00*
60–69	1.23 (0.91–1.66)		1.21 (0.73–2.01)		1.2 (0.8–1.81)	
70+	2.38 (1.66–3.4)		2.32 (1.34–4.00)		2.46 (1.49–4.05)	
BMI						
Normal (reference)	1	0.03*	1	0.97	1	0.06
Low	1.71 (0.85–3.43)		0.92 (0.38–2.23)		3.16 (1.06–9.36)	
Obesity	1.36 (1.05–1.76)		1.03 (0.64–1.64)		1.6 (1.16–2.22)	
Educational level						
College (reference)	1	0.00*	1	0.00*	1	0.00*
Elementary	5.66 (3.36–9.53)		6.33 (3.44–11.65)		3.94 (1.51–10.29)	
Middle	3.15 (1.88–5.3)		3.14 (1.54–6.42)		2.64 (1.05–6.62)	
High	2.75 (1.63–4.64)		3.43 (1.6–7.36)		1.67 (0.64–4.38)	
Household net income						
High (reference)	1	0.00*	1	0.02*	1	0.00*
Low	1.76 (1.25–2.49)		1.04 (0.62–1.75)		2.8 (1.85–4.24)	
Low to mid	1.23 (0.83–1.84)		0.97 (0.51–1.86)		1.6 (1.01–2.53)	
Mid to high	0.86 (0.58–1.27)		0.49 (0.28–0.86)		1.24 (0.77–2)	
Residential area						
City (reference)	1	0.02*	1	0.01*	1	0.50
Rural	1.37 (1.06–1.78)		1.83 (1.15–2.91)		1.12 (0.81–1.55)	
Current marital status						
Married (reference)	1	0.63	1	0.43	1	0.52
Unmarried, divorced, widow or widower	1.08 (0.79–1.48)		0.78 (0.41–1.45)		1.13 (0.78–1.64)	
Employed						
Yes (reference)	1	0.01*	1	0.17	1	0.01*
No	1.47 (1.1–1.96)		1.32 (0.89–1.96)		1.62 (1.14–2.31)	
Smoking						
No (reference)	1	0.80	1	0.90	1	0.56
Yes	1.06 (0.68–1.66)		1.03 (0.62–1.73)		1.31 (0.52–3.29)	
Diabetes						
No (reference)	1	0.19	1	0.15	1	0.71
Yes	1.17 (0.93–1.48)		1.28 (0.92–1.8)		1.07 (0.75–1.52)	
Hypertension						
No (reference)	1	0.27	1	0.68	1	0.11
Yes	1.18 (0.88–1.58)		0.92 (0.62–1.37)		1.4 (0.92–2.12)	
CAD						
No (reference)	1	0.09	1	0.81	1	0.03*
Yes	1.54 (0.93–2.54)		0.93 (0.51–1.7)		2.48 (1.11–5.51)	
Dyslipidaemia						
No (reference)	1	0.21	1	0.80	1	0.22
Yes	1.26 (0.88–1.8)		1.1 (0.54–2.21)		1.31 (0.85–2.01)	
Stroke						
No (reference)	1	0.05	1	0.04*	1	0.82
Yes	2.03 (1–4.13)		2.75 (1.03–7.37)		1.07 (0.6–1.9)	
CRD						
No (reference)	1	0.91	1	0.07	1	0.03*
Yes	1.2 (0.05–27.05)		9.44 (0.8–111.3)		0.19 (0.04–0.82)	
Arthritis						
No (reference)	1	0.00*	1	0.00*	1	0.00*
Yes	4.03 (3.19–5.09)		3.3 (2.16–5.05)		4.58 (3.34–6.27)	
Cancer						
No (reference)	1	0.02*	1	0.06	1	0.18
Yes	1.48 (1.08–2.05)		1.68 (0.98–2.88)		1.34 (0.87–2.07)	
Depression						
No (reference)	1	0.00*	1	0.00*	1	0.00*
Yes	2.71 (1.96–3.75)		3.28 (1.81–5.96)		2.67 (1.9–3.76)	
R^2^	0.41	0.29			0.43	

*P < 0.05

In adults aged over 50 years, the following factors had significant negative associations with the EQ-5D index score (*P* < 0.05): ageing (odds ratio (OR) (95% confidence interval), age 60–69 group: 1.23 (0.91–1.66), age 70 group: 2.38 (1.66–3.4)); abnormal BMI (low: 1.71 (0.85–3.43), obesity: 1.36 (1.05–1.76)); lower educational level (elementary: 5.66 (3.36–9.53), middle: 3.15 (1.88–5.3), high: 2.75 (1.63–4.64)); lower household net income (low: 1.76 (1.25–2.49), low to mid: 1.23 (0.83–1.84)); rural residential area (OR: 1.37 (1.06–1.78)), unemployed status (OR: 1.47 (1.1–1.96)); arthritis (OR: 4.03 (3.19–5.09)); cancer (OR: 1.48 (1.08–2.05)); and depression (OR: 2.71 (1.96–3.75)). However, gender had no significant association with the EQ-5D index score.

In the male group, age, educational level, household net income, residential area, stroke, arthritis, and depression had significant negative associations with the EQ-5D index score (*P* < 0.05). In the female group, age, educational level, household net income, employed status, CAD, CRD, arthritis, and depression had significant associations with the EQ-5D dimensions index score (*P* < 0.05). In both genders, both arthritis (OR: 3.3 (95% CI: 2.16–5.05) in the male group, 4.58 (3.34–6.27) in the female group) and depression (3.28 (1.81–5.96) in the male group, 2.67 (1.9–3.76) in the female group) had significant negative associations with the EQ-5D index score (*P* < 0.05) (see details in Table 4).

## Discussion

Our results demonstrated that age, socio-economic status, arthritis and depression were the most important factors associated with health status (see Tables 3, 4).

The EQ-5D index scores were estimated as 0.81 ± 0.03 in males and 0.81 ± 0.03 in females. Gender did not affect EQ-5D index scores after adjusting for all covariates, and there were also no significant differences in EQ-5D index scores between genders when stratified by age groups. This result was consistent with those of previous studies of older adults in Vietnam [15] and rural elderly individuals in Egypt [16] but was not in line with the result that women had lower EQ-5D index scores in other countries [17–19]. The EQ-5D instrument asks about the domains of QoL as they are experienced on just 1 day, so it may be insufficient to determine QoL related to conditions that are intermittently symptomatic [20]. Therefore, these findings may suggest that gender-specific disease symptoms or conditions are not precisely reflected by available QoL measuring tools.

In present study, after controlling for the covariates, age over 70 years, lower educational level (elementary < middle and high < college in the male group; elementary < middle < high and college in the female group) and household net income (low < low to mid and high < mid to high quartiles in the male group, low < low to mid < mid to high < high quartiles in the female group) were associated with lowering the EQ-5D index in both genders. Low socio-economic status may place individuals at risk for poorer health states for a variety of reasons, such as having less access to healthcare, poorer living conditions, less knowledge about the negative consequences of health-compromising behaviours, and greater psychological stress [21, 22].

The interesting finding of our results was that among underlying medical comorbidities, arthritis and depression had negative associations with health status (see Tables 3 and 4), although stroke, chronic renal disease and cancer, in association with lowering the EQ-5D index, showed different results according to gender or ageing.

The prevalence of arthritis was higher in females (40.2%) than in males (13.6%) and was the third-highest after HBP and DM, and women had a higher proportion of depression (22.8%) than men (9.3%) in our results.

Arthritis is the greatest cause of chronic pain and functional disability among older people. According to a cross-sectional study in the United States, there were significant associations between arthritis symptom clusters and both QoL and functional status [23]. It was also reported that women with OA had poorer scores compared to men for bodily pain, general health, and mental health after adjusting for age and disease severity, and low educational attainment was independently associated with poorer scores [24].

Depression was also reported to be a factor having a negative association with QoL [25]. Depression was correlated with the physical and psychological components of the QoL questionnaire in chronic diseases [26, 27]. The prevalence of depression increases more frequently among older people than among younger people [28].

Chronic diseases themselves are reported to be an important contributing factor in lowering HR-QoL [18]. Typically, DM [29] and cancer [30] are known to be important factors in HR-QoL because they could cause multiple complications that lead to functional decrements affecting HR-QoL. However, our results showed that DM, HBP, CAD and dyslipidaemia had no significant associations with HR-QoL, after adjusting for all independent variables. Stroke had no association with HR-QoL, and CRD and cancer had associations with HR-QoL in only the male group when findings were stratified by gender.

These interesting results may suggest several possible reasons for the weak association between these medical comorbidities and health status. First, the impact of these chronic medical conditions on HR-QoL could vary, as a large spectrum of severity exists in each disease. Second, the Korean national health insurance system covers the entire population living in the country, and hospitals are very well equipped. According to the 2015 health data provided by the Ministry of Health and Welfare and the OECD, Korean individuals visited the doctor’s office an average of 14.6 times in 2013, which is the highest among OECD member countries. The characteristics of the Korean medical system may create a much lower burden for the management of chronic diseases and contribute to different results from other countries.

Despite the fact that arthritis and depression are modifiable factors supported by the medical system, they had negative associations with health status, regardless of age or gender. This finding may suggest that arthritis and depression have distinct characteristics affecting HR-QoL, unlike other chronic diseases. Previous studies [31, 32] have indicated that high functional ability or leisure and vocational activities are associated with lowering depression levels, and the frequency of leisure activities contributes to preventing depression in noninstitutionalized older adults with cerebrovascular diseases. Consistent with these past studies, our results may suggest that older people place more importance on the ability to perform daily or social activities in a healthy state than whether they have a chronic disease. Additionally, relationships with family, relatives or friends in daily activities may lead to a feeling of being healthy.

Therefore, our study suggests that subjective feelings, such as the emotions or self-esteem built through daily activities or relationships with other people, which could be easily overlooked, may be important factors associated with HR-QoL as well as medical comorbidities and socio-economic status, which could be supported by the medical and social welfare system.

However, our study has limitations in explaining the causal relationship between multiple factors, such as arthritis/depression and QOL, because our study is cross-sectional in design. Second, there may be the possibility of sample selection bias and endogeneity bias, although we analysed the data using sampling weights, considering dropped data and the non-response rate. Third, we also tried to include as many independent variables we could, but there may still be other disease categories associated with QOL.

## Conclusions

Ageing and low socio-economic status were the primary factors associated with low self-rated health status. Among chronic diseases that have been reported to be associated with QOL in previous reports, arthritis and depression, which could be modifiable factors, were the main factors associated with health status in older people. This finding suggests that it may be necessary to pay more attention to the factors associated with musculoskeletal pain, emotional distress, socio-economic status and chronic diseases.

## Abbreviations

CAD: Coronary artery disease
CI: Confidence interval
CRD: Chronic renal disease
DM: Diabetes mellitus
EQ-5D: EuroQol 5-Dimension Questionnaire
HBP: Hypertension
HR-QoL: Health-related quality of life
KCDC: Korea Centers for Disease Control and Prevention
KNHANES: Korea National Health and Nutrition Examination Survey
QoL: Quality of life
SE: Standard error
SF-6D: Short Form-6 dimension
WHO: World Health Organization
